# P-662. Phase III, modified double-blind, randomized, parallel group, active-controlled, multi-center study of Meningococcal quadrivalent ACWY conjugated vaccine in infants from 6 through 23 months of age in the United States

**DOI:** 10.1093/ofid/ofae631.859

**Published:** 2025-01-29

**Authors:** Cheryl Duffy, Betzana Zambrano, Mandeep S Dhingra, Olga Lyabis, Julie Chaix, Siham Bchir, Sandeep Gupta, Christine Rehm

**Affiliations:** Kids Way Clinic, Hermitage, Pennsylvania; Sanofi Pasteur, Montevideo, Montevideo, Uruguay; Sanofi Pasteur, Montevideo, Montevideo, Uruguay; Sanofi, Marcy L’ Etoile, Auvergne, France; Sanofi, Marcy L’ Etoile, Auvergne, France; Sanofi, Marcy L’ Etoile, Auvergne, France; Sanofi, Marcy L’ Etoile, Auvergne, France; Sanofi, Marcy L’ Etoile, Auvergne, France

## Abstract

**Background:**

Invasive meningococcal disease (IMD) occurs worldwide in both endemic and epidemic forms. In the US in 2022, the incidence of invasive meningococcal disease (IMD) was 0.09 per 100,000 population, with the highest rates observed in infants < 1 year of age. MenQuadfi^®^ (MenACYW conjugate vaccine) is currently licensed in the US against IMD for individuals 2 years and older and is under development for use from 6 weeks of age to provide protection for all age groups against IMD caused by serogroups A, C, Y and W.

**Figure:**
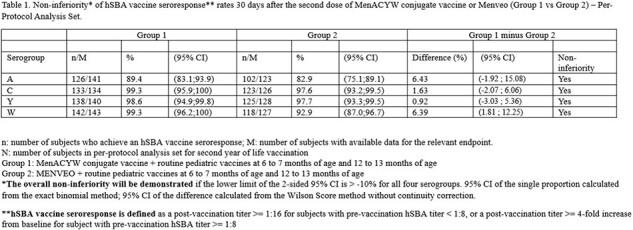

**Methods:**

This was a phase III study (NCT03691610) performed to evaluate the immunogenicity and safety of MenACYW conjugate vaccine (Group 1) and Menveo^®^ (Group 2) when administered in a 2-dose schedule concomitantly with routine pediatric vaccines at 6 -7 and 12 – 13 months of age, and to describe the safety and immunogenicity of MenACYW conjugate vaccine (Group 3) and Menactra^®^ (Group 4) when administered in a 2-dose schedule at 17 – 19 and 20 – 23 months of age. The primary objective of the study was to demonstrate non-inferiority of the MenACYW conjugate vaccine seroresponse as measured by serum bactericidal assay using human complement (hSBA) to serogroups A, C, W and Y when given at 6-7 and 12-13 months of age compared to that of Menveo^®^.

**Figure:**
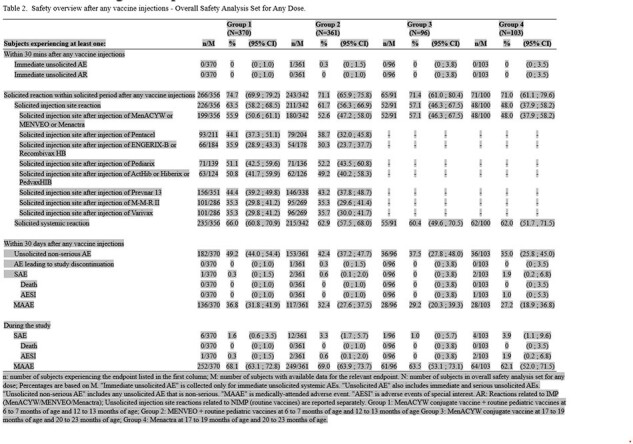

**Results:**

950 participants were enrolled; 452 (47.6%) were female, 160 (16.8%) were Black or African American, 15 (1.6%) were Asian origin, and 396 (41.7%) were Hispanic or Latino. The primary objective of the study was met: the percentage of subjects who achieved a vaccine seroresponse post-dose 2 for all four meningococcal serogroups in Group 1 was 89.4% - 99.3% depending on serogroup and was non-inferior to the corresponding percentage seen in Group 2 (Table 1). The safety profile of MenACYW conjugate vaccine was comparable to Menveo^®^ when both were administered concomitantly with routine pediatric vaccines at 6 – 7 and 12 – 13 months of age. The safety profiles of MenACYW conjugate vaccine and Menactra^®^ when administered to older toddlers were also comparable (Table 2).

**Conclusion:**

MenACYW conjugate vaccine is immunogenic and demonstrates an acceptable safety profile when administered to infants 6 through 23 months of age as a 2-dose schedule with a minimum interval of 3 months.

**Disclosures:**

**Cheryl Duffy, MD**, Sanofi: Support for current manuscript, Investigator Meeting attendance for this study **Betzana Zambrano, MD**, Sanofi: Employee|Sanofi: Stocks/Bonds (Public Company) **Mandeep S. Dhingra, MD**, Sanofi: Employee|Sanofi: Stocks/Bonds (Public Company) **Olga Lyabis, MD**, Sanofi: Employee|Sanofi: Stocks/Bonds (Public Company) **Julie Chaix, n/a**, Sanofi: Employee **Siham Bchir, MSc**, Sanofi: Employee|Sanofi: Stocks/Bonds (Public Company) **Sandeep Gupta, MD**, Sanofi: Employee|Sanofi: Stocks/Bonds (Public Company) **Christine Rehm, MD**, Sanofi: Employee|Sanofi: Stocks/Bonds (Public Company)

